# Juvenile Recurrent Parotitis: An Eight-Year-Old Boy With a Painful Acute Right-Sided Parotid Swelling

**DOI:** 10.7759/cureus.42632

**Published:** 2023-07-28

**Authors:** Meshael M Alkusayer, Reham S Alshehri, Reema S Alkhoudairy, Rawan M Alahmadi, Abdullah A Albdah

**Affiliations:** 1 Otolaryngology, Head and Neck Surgery, King Saud Medical City, Riyadh, SAU; 2 College of Medicine, Princess Nourahbint Abdulrahman University, Riyadh, SAU; 3 Otolaryngology, Head and Neck Surgery, Prince Sultan Military Medical City, Riyadh, SAU

**Keywords:** lymphadenopathy, ultrasonography, saudi arabia, parotid gland, parotitis

## Abstract

Juvenile recurrent parotitis (JRP) is a rare recurrent non-obstructive inflammatory swelling of the parotid gland occurring most commonly in children aged three to six years. JRP is usually idiopathic and presents as a painful swelling recurring on either side of the face at least twice within six months. We report the case of an eight-year-old Saudi boy with a painful acute right-sided parotid swelling and a history of similar occurrences bilaterally at least four times a year for two years. The routine laboratory investigations were unremarkable. Ultrasonography of the parotid glands suggested parotitis with cervical lymphadenopathy. He was treated conservatively and remained asymptomatic for a year. Although rare, an accurate diagnosis of JRP is possible with adequate history, physical examination, and lab investigations, supplemented with radiographic findings.

## Introduction

Juvenile recurrent parotitis (JRP) is defined as recurrent non-obstructive non-neoplastic inflammation of the parotid gland [1]. JRP is the second most common cause of parotitis among children [1]. A rare condition with unknown etiology, it usually occurs at the age of three to six years, and most symptoms resolve after puberty [2]. During an acute exacerbation, the patient presents with swelling and/or pain associated with fever or malaise [3]. All suspected JRP patients should be screened to exclude immunodeficiency, human immunodeficiency virus (HIV), sarcoidosis, and Sjögren’s syndrome to ascertain the correct diagnosis [4].

## Case presentation

An eight-year-old Saudi boy presented to the pediatric otorhinolaryngology clinic complaining of right-sided facial swelling for five days. The swelling was mainly in the parotid area and the cheek and was associated with pain, discomfort, and mild erythema over the skin. The patient reported adequate oral hygiene with normal salivary status. There was no history of ear infections and facial nerve function was intact. Otoscopic examination showed normal looking intact tympanic bilaterally.

**Figure 1:**
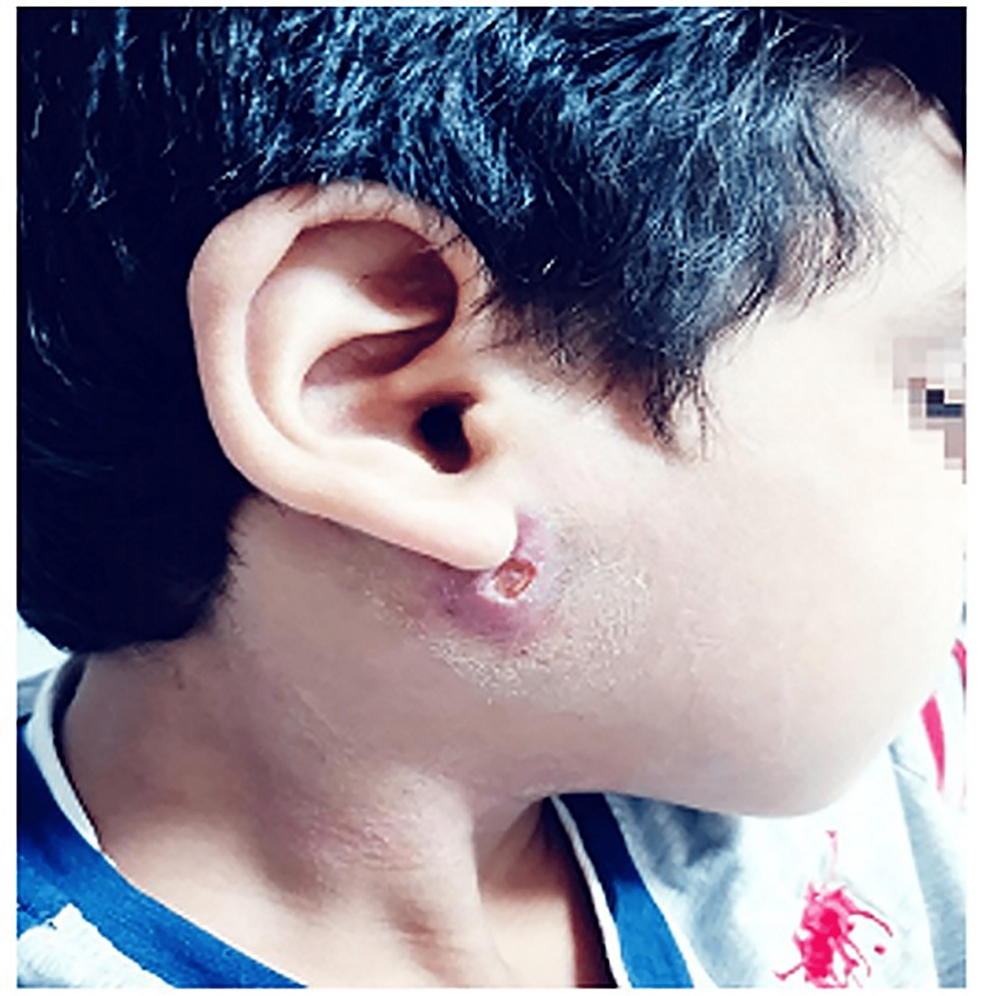
Clinical photograph of an eight-year-old boy with right-sided facial swelling. A hypertrophic scar was noticed post extraoral incision and drainage.

When firm pressure was applied over the gland, no purulent discharge was noticed from the parotid duct. However, a hypertrophic scar was noticed in front of the tragus on the right side from an extraoral incision and drainage for the same condition performed three months before the clinic visit in another hospital (Figure 1).

The patient reportedly had recurrent swellings on either side of the face alternately from two years of age, at least four times a year. The symptoms subsided on taking antibiotics and analgesics sometimes, while mostly they resolved spontaneously. During these episodes, the patient did not experience any dryness of mouth, increase in salivary production, or taste alteration. The patient remained otherwise healthy with no relevant medical history or family history of autoimmune disorders. His immunization status was also up to date as per the Saudi immunization program.

Routine laboratory examinations, erythrocyte sedimentation rate (ESR), and serum immunoglobulin levels were within normal limits. HIV serology, Sjögren antibodies, and rheumatoid factor were negative. Ultrasonography of the bilateral parotid glands done two weeks prior to presentation was suggestive of bilateral parotitis with bilateral cervical lymphadenopathy. A previous right parotid gland core needle biopsy favored the diagnosis of sialosis (sialoadenosis).

**Figure 2:**
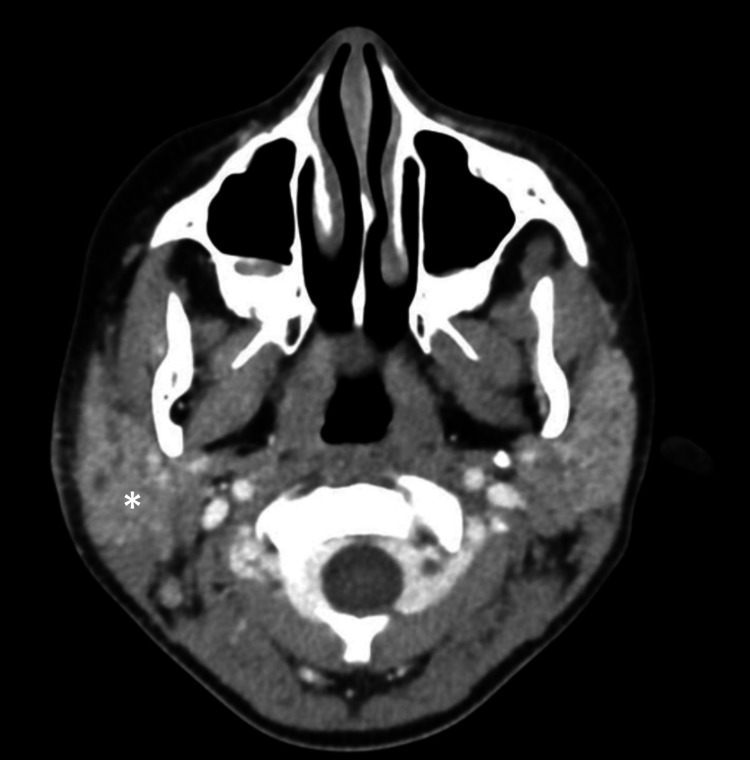
CT scan with contrast of the head and neck showing right parotitis with adjacent soft tissue edema and reactive lymphadenopathy (*)

**Figure 3:**
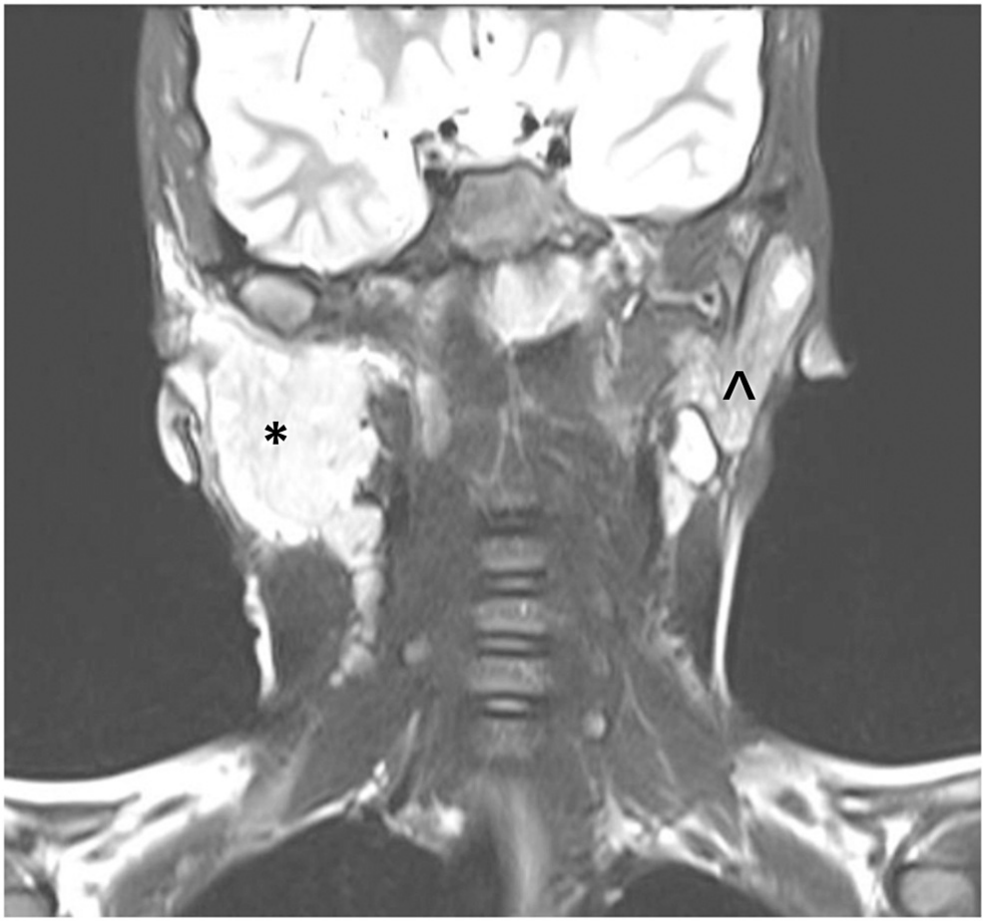
MRI of the patient reviling a right acute-on-chronic parotiditis (*) and chronic left parotiditis with mild atrophic changes (^)

Computed tomography (CT) scan and magnetic resonance imaging (MRI) were performed within a week of the clinic visit which revealed right acute-on-chronic parotiditis (sialadenitis) with adjacent soft-tissue edema, and reactive lymphadenopathy with chronic left parotiditis with mild atrophic changes (Figures 2, 3).

**Figure 4:**
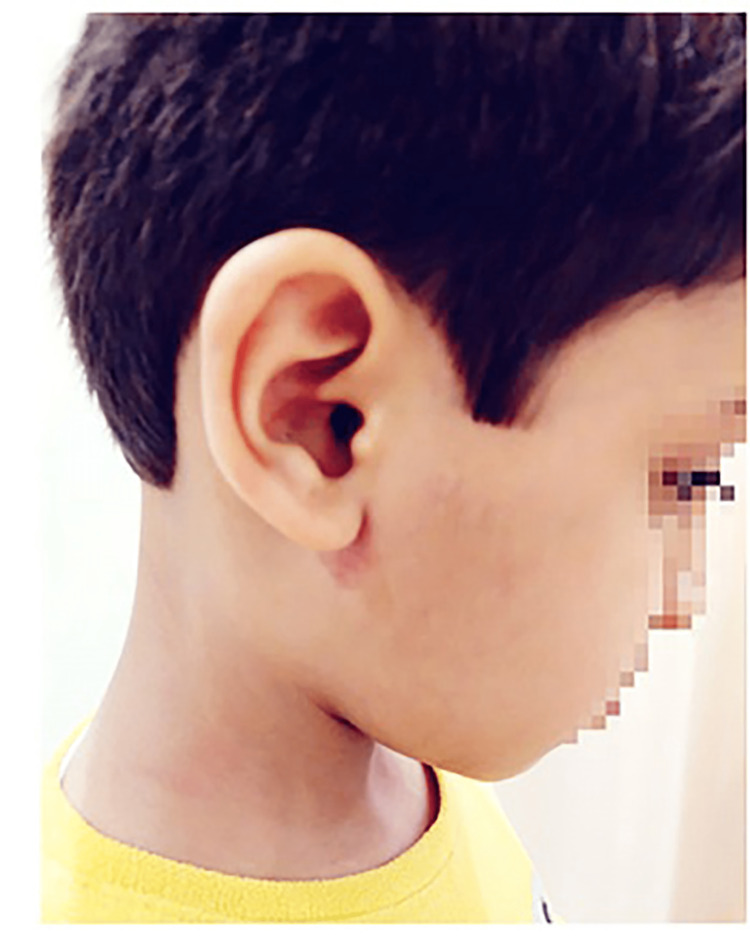
Clinical photograph six months after the initial presentation.

The patient was treated conservatively with pain-relieving medications with warm compresses and monitored closely in subsequent follow-ups at the clinic. He remained asymptomatic for the next 18 months (Figure 4).

## Discussion

JRP can be diagnosed clinically with appropriate history and physical examination to rule out other diseases. However, the clinician must necessarily suspect JRP as a differential diagnosis since the clinical presentation usually resembles mumps in the index episode [5]. Generally, JRP presents as a recurrent unilateral swelling of the parotid gland associated with pain, fever, and malaise that persists between one to 14 days [5]. Therefore, the duration of symptoms and frequency of attacks is important to diagnose the condition. Moreover, excluding a history of previous trauma, dental or other surgical procedures in this area, and other infections is vital for the diagnosis.

The clinician must inspect for any skin changes, signs of dehydration, or facial asymmetry in the physical examination. The parotid gland and facial nerve should be examined simultaneously [4,6].

Patients suspected of JRP are suggested to undergo basic laboratory tests, biochemical analysis, immunological tests, and serological tests to exclude immunodeficiency, HIV infection, and autoimmune diseases like sarcoidosis and Sjögren’s syndrome [6]. These tests include complete blood count, ESR, C-reactive protein levels, and serological tests for mumps and other infectious diseases that may cause parotid swelling. Additionally, serum complement component 3 and 4 levels, immunoglobulin levels, antinuclear antibodies (ANA), anti-double-stranded deoxyribonucleic acid antibodies (anti-dsDNA) for systematic lupus erythematosus, and SS-A/anti-Ro, SS-B/anti-La antibodies for Sjögren’s syndrome [4].

Ultrasound sialography is considered an essential imaging method for diagnosing and describing the ductal system in JRP patients [4,6]. However, ultrasound is a non-invasive, cheap, and quick imaging modality for diagnostic and follow-up purposes while assessing for JRP. Ultrasonography in JRP shows sialectasis that appears as intraductal cystic dilation surrounded by lymphatic infiltrate, whereas sialography demonstrates sialectasis with no ductal obstruction [7,8]. Additionally, sialendoscopy is performed to diagnose and treat JRP patients. The typical findings during visualizing the duct using an endoscope are plugging and scarring of the mucosa with ductal stenosis [9].

Recently, Garavello et al. [6] suggested a set of criteria to diagnose JRP. The inclusion criteria describe a patient aged <16 years with unilateral or bilateral parotid gland swelling and at least two episodes during the last six months. They further stated that conditions like obstructive lesions of the parotid glands, Sjögren’s syndrome, dental malocclusion, and congenital IgA immunodeficiency must be excluded before diagnosing JRP.

In general, a combination of the patient’s history (recurrent unilateral swelling of the parotid gland associated with pain, fever, and malaise that persists up to two weeks), examination, and laboratory findings have significant diagnostic value, which can be further confirmed by radiological findings [5].

The management of JRP can be either conservative or surgical treatment [10,11]. The initial conservative treatment is symptomatic, which includes high fluid intake, warm compression, parotid massage, and the use of antibiotics and analgesics (such as non-steroidal anti-inflammatory drugs) [12-15]. Patients in whom conservative treatment has failed are potential candidates for surgical intervention [16]. A sialoendoscopy without corticosteroid has increasingly become the first-line treatment since it has shown a remarkable improvement with a concomitant decrease in the frequency of attacks in JRP patients [17,18]. A retrospective study of 110 JRP patients highlighted the role of sialography in evaluating and treating the affected cases. Following sialography, 89% of the patients showed a marked improvement in the disease course [19]. Additionally, Roby et al. stated that a dose of 100 mg hydrocortisone ductal infusion through catheter inserted in the parotid duct alone has a beneficial role in reducing the incidence of the attacks [8]. 

Another case series suggested that a new alternative treatment method using saline irrigation of the parotid duct by siloendoscopy without anesthesia is less invasive and has no side effects [18]. Total resolution of symptoms was observed among two children and improvement in condition was observed in the rest of the four children. Throughout the study, no noticeable side effects were observed (Table 1).

**Table 1:** A review of 101 patients presenting with juvenile recurrent parotitis along with the different treatment modalities used. NA = no data available, Cured = patients who had complete symptoms resolution, Improved = patients who had reduced the frequency of attacks

Reference	Case details				Episode Details		Management	Treatment Outcome		Follow-up (months)	
	No. of cases	Gender	Age at the first attack (year)	Gland site	Duration (month)	Frequency (year)		Cured	Improved		
Rao et al. [10]	1	Male	5	Right	NA	8-9/year	Amoxicillin. Clavulanic acid, analgesic, chewable tablet	1		24	
Sahin et al. [11]	1	Male	3	Bilateral	7-10 days	5/year	Ibuprofen, ampicillin-sulbactam, and fluid intake		1	6	
											Reddy et al. [12]	1	Male	1	Bilateral	NA	Twice/year	Analgesic and antibiotic	1		18	
Singh et al. [13]	3	Male (1) Female (2)	1.5-2	Bilateral	NA	4-10 episodes	Antibiotic, analgesic, parotid massage, chewing gum	2	1	12												
Tomar et al. [14]	1	Male	5	Bilateral	8-10 days	4 years	High fluid intake and analgesic	cured		18	
											Almeshary et al. [15]	1	Female	11	Left	4-7 days	10 episodes in 2 years	High fluid intake, analgesic, and parotid massage	cured		22	
Nouha et al. [16]	1	Male	4	Left	5 days	First episodes	Systemic antibiotic	cured		4	
Alataki et al. [17]	23	12- Males 11- Females	Mean 3.5 years	10-Right 7-Left 6-Bilateral	NA	2-8/year	Primarily with conservative therapy then 12 cases undergo sialoendoscopy		All improved	48	
Geisthoff et al. [18]	6	Male	1.2 – 6.2 (mean 3.7 years)	5-Right 1-Left	Mean 6.4	4-12/year	Irrigation with saline solution	4	2	27.7	
Benaim et al. [19]	41	22- Males 19- Females	Mean 6.7 years	Bilateral/unilateral	>2	≥6 months	Antibiotics, analgesics and steroids then sialoendoscopy	75% underwent sialoendoscopy and cured	25% improved with antibiotics, analgesics and steroid		
Faizal et al. [20]	22	14- Males 8- Females		Bilateral/unilateral	>2	≥6 months	Antibiotics, analgesics and steroids then sialoendoscopy	All cured		NA	

Benaim et al. [20] conducted a retrospective study for a period of 10 years which included 41 patients suffering from JRP to evaluate the treatment outcomes and provide a better way for disease management based on evidence. They found two treatment strategies in their selected cohort, viz. antibiotics and sialoendoscopy. It was observed that three-fourths of the patients who underwent sialoendoscopy for treatment did not show any recurrence of the disease after three sialoendoscopies. The study concluded that clindamycin could be an effective antibiotic for initial treatment and severe recurrence of the disease could be controlled by sialoendoscopy (Table 1). Sialoendoscopy is being used alone for the treatment of JRP or used along with ductal corticosteroid infusion (DCI). Both methods have shown effectiveness in the treatment of JRP [21]. A study including 12 patients was conducted to evaluate whether DCI alone was effective in the treatment of JRP. These patients were treated with DCI using a catheter for DCI with hydrocortisone. Treatment with DCI alone showed improvement in patients with increase in time-duration of disease recurrence. This study showed that DCI alone can treat JRP patients and showed similar results as sialoendoscopy along with corticosteroid, indicating that it was the application of corticosteroids that caused the improvement in the disease symptoms [21]. Faizal et al. in their study of 22 patients found that sialoendoscopy showed better results in these patients who were managed medically prior to salivary endoscopy in terms of pain scores and longer remission time (Table 1) [22]. 

Overall, the data suggest that sialoendoscopy is a better treatment option for JRP with or without corticosteroids.

## Conclusions

JRP is a treatable condition that can be appropriately managed conservatively when the diagnosis is made timely. The clinician must always consider JRP as a differential diagnosis when treating a pediatric patient who presents with recurrent parotid swelling and associated pain, fever and malaise. The attacks occur more than two times in a period of six months and last for three to five days up to two weeks. Judicious use of laboratory investigations and serological analysis to exclude other causes of parotid swelling, supplemented with ultrasonography, may help diagnose the condition accurately. In the case of failed conservative management, sialoendoscopy without corticosteroids and with intraductal saline irrigation can be successfully used to treat JRP and reduce recurrences.
